# Coronavirus takeover of host cell translation and intracellular antiviral response: a molecular perspective

**DOI:** 10.1038/s44318-023-00019-8

**Published:** 2024-01-10

**Authors:** Evangelos D Karousis, Katharina Schubert, Nenad Ban

**Affiliations:** 1 Multidisciplinary Center for Infectious Diseases, University of Bern, Bern, Switzerland; 2 Department of Chemistry and Biochemistry, University of Bern, Bern, Switzerland; 3 Department of Biology, Institute of Molecular Biology and Biophysics, ETH Zurich, Zurich, Switzerland

**Keywords:** Coronaviruses, Viral protein synthesis, SARS-CoV-2, Translation, Translation regulation, Microbiology, Virology & Host Pathogen Interaction, RNA Biology

## Abstract

Coronaviruses are a group of related RNA viruses that cause respiratory diseases in humans and animals. Understanding the mechanisms of translation regulation during coronaviral infections is critical for developing antiviral therapies and preventing viral spread. Translation of the viral single-stranded RNA genome in the host cell cytoplasm is an essential step in the life cycle of coronaviruses, which affects the cellular mRNA translation landscape in many ways. Here we discuss various viral strategies of translation control, including how members of the *Betacoronavirus* genus shut down host cell translation and suppress host innate immune functions, as well as the role of the viral non-structural protein 1 (Nsp1) in the process. We also outline the fate of viral RNA, considering stress response mechanisms triggered in infected cells, and describe how unique viral RNA features contribute to programmed ribosomal −1 frameshifting, RNA editing, and translation shutdown evasion.

## Abstract (web summary)

This review discusses the unique features of coronaviruses that enable them to commandeer host cell translation while avoiding host immune defense responses.

## Graphical abstract

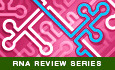

## Introduction

Protein synthesis is the last stage of expressing genetic information, during which the information stored in messenger RNA (mRNA) is decoded. mRNA translation is constantly monitored by extensive cellular machinery responsible for mRNA and nascent protein quality control (Joazeiro, 2019). Due to the importance of translation, these cellular processes also provide an evaluation of the cellular metabolic and functional state (Yip and Shao, 2021). Consequently, since viruses rely on host translation to produce proteins, their perturbation of protein synthesis in infected cells impacts all aspects of cellular function.

Coronaviruses are positive-sense single-stranded RNA viruses because their RNA genome can be directly translated into proteins. They infect animals and humans, and, like many other viruses, have evolved an arsenal of mechanisms to bypass cellular innate immune mechanisms which detect viral infections and prevent foreign RNA translation (V’kovski et al, 2021; de Breyne et al, 2020). In general, viral RNA genomes are compact and rely on various strategies to extend their coding capacity. Furthermore, they have adapted to the monocistronic nature of eukaryotic translation that depends on 5΄ cap recognition. RNA viruses often rely on translation of polyproteins that are cleaved by viral proteases into multiple functional units. By employing leaky scanning, ribosomal frameshifting, reinitiation, and readthrough, they produce multiple proteins from one genome. Coronaviruses employ most, if not all, of these strategies to hijack the host cell protein synthesis and ensure production of viral proteins. The importance of translational control in the life cycle of coronaviruses is evident from the observation that the first protein produced upon *Betacoronavirus* (β-CoV) infections inhibits host translation (Hartenian et al, 2020; Schubert et al, 2020; Thoms et al, 2020). Upon infection, the proteome is reshaped due to Nonstructural protein 1 (Nsp1) promoting coronaviral mRNA translation and downregulating host translation (de Breyne et al, 2020).

Recent advancements in biophysical and molecular biology techniques have contributed to our understanding of how coronaviruses take over host protein synthesis and how the cell responds to the viral infection. Here we present recent insights obtained using cryo-electron microscopy (cryoEM) to reveal high-resolution structures of cellular complexes (Saibil, 2022), ribosome profiling to map locations of translating ribosomes on mRNAs (Ingolia et al, 2019), single-molecule Förster resonance energy transfer (smFRET) experiments to obtain information about the dynamics of the participating molecular complexes in real time (Prabhakar et al, 2019), and mass spectrometry (mass-spec) to monitor the landscape of protein changes and interactions in infected cells (Iwasaki and Ingolia, 2017).

This review provides an overview of how coronaviruses control viral and host translation during different stages of infection and compares these strategies to those of other RNA viruses. We focus on how Nsp1 from β-CoV hijacks the translation machinery and describe viral RNA features that promote viral evasion and programmed ribosomal frameshifting (PRF) in infected cells. Furthermore, we discuss other unique features of viral RNA and various stress response mechanisms triggered by coronaviral infection.

## Basic features of human translation and translation regulation

In eukaryotic cells, the canonical translation process can be divided into four main stages: initiation, elongation, termination, and recycling. During initiation, more than 12 initiation factors participate in binding the mRNA, scanning, and identification of the start codon. This process entails a coordinated series of conformational changes and exchange of initiation factors on the small ribosomal subunit. Initially, a ternary complex of Met-tRNA_i_^Met^ and eukaryotic initiation factor 2 (eIF2-GTP), eIF3, eIF1A, eIF1 and eIF5 bind the small ribosomal subunit (40S), forming the 43S pre-initiation complex (PIC). The multi-subunit eIF3 complex plays a particularly important role throughout initiation by coordinating the binding of other initiation factors and interacting with eIF1 and the cap-binding multi-subunit complex eIF4F. Once the 43S PIC binds to the mRNA and eIF4F, the resulting 48S PIC scans the mRNA until it encounters a start codon (Shirokikh and Preiss, 2018; Brito Querido et al, 2020). At the conclusion of the initiation stage, an elongation competent 80S initiation complex is formed, with Met-tRNA^Met^ bound to the start codon in the peptidyl-site (P-site) of the ribosome. During elongation, the polypeptide chain is synthesized through the sequential addition of amino acids delivered by aminoacyl-tRNAs (aa-tRNA), as specified by the codons in the open-reading-frame (ORF) of the mRNA. Dedicated elongation factors (eEFs) deliver the tRNAs and translocate the ribosome along the mRNA. Translation terminates when the ribosome encounters a stop codon that cannot be recognized by any of the aa-tRNAs. Specific eukaryotic release factors (eRF1 and eRF3) recognize the termination codons and promote peptide release. The ribosome disassembles and dissociates from the mRNA, aided by the recycling factor ABCE1 (for a review of the translational cycle in eukaryotes, see Dever and Green, 2012).

Regulation of translation occurs primarily at the level of translation initiation. One mechanism that results in protein synthesis inhibition, referred to as the integrated stress response (ISR), targets initiation factor eIF2 in response to different forms of stress, such as amino acid deprivation, oxidative stress, endoplasmic reticulum (ER) stress or viral infections. During the ISR, the pool of available eIF2-GTP is reduced, thereby reducing the availability of 43S complexes. Four different Ser/Thr kinases, each activated by different stress cues, can lead to global protein synthesis inhibition by phosphorylating the eIF2α subunit. For example, in response to the dsRNA presence in the cell that often signifies viral infections, double-stranded RNA-dependent protein kinase (PKR) is activated, whereas general control nonderepressible 2 (GCN2) kinase is activated during amino acid deprivation (Roux and Topisirovic, 2012). Phosphorylation of eIF2α leads to its tight binding to eIF2B and does not allow the exchange of GDP with GTP, rendering eIF2 unavailable for subsequent rounds of translation. Already small changes in eIF2α phosphorylation can quickly cause global translation inhibition (Costa-Mattioli and Walter, 2020; Burgess et al, 2022). eIF2α phosphorylation also stimulates the formation of cytoplasmic foci called stress granules, which contain mRNAs stalled at translation initiation, RNA binding proteins, translation factors, and the 40S subunits. However, although cellular stress leads to a general translation shutdown, specific mRNA populations can nevertheless be selectively translated (Schuller and Green, 2018).

The kinetic status of translation acts as a quality control sensor. In general, ribosomal stalling, frequently accompanied by ribosome collisions, is linked to RNA and protein quality control processes that probe and degrade defective molecules (D’Orazio and Green, 2021). In extreme cases, ribosome stalling can activate transcriptional changes and even induce cell apoptosis (D’Orazio and Green, 2021).

## Viral strategies to hijack host cell translation

Since viruses rely on host cellular components and molecular machines to replicate and translate their genomes, they evolved many different strategies to control the cellular translation machinery. Viruses target different stages of translation and interfere with cellular antiviral response pathways through the production of viral proteins or due to the unique features of their single-stranded RNA molecules that can adopt functional folds. One of the main determinants of how RNA viruses interact with the translation machinery is the characteristics of the viral genome, namely, whether it is a positive or negative sense single-stranded RNA (ssRNA) or a double-stranded RNA (dsRNA). Positive-sense ssRNA viruses directly use their own genome as an mRNA to produce polyproteins (Modrow et al, 2013). In contrast, negative sense ssRNAs genomes enter the cell together with a viral RNA-dependent RNA polymerase (RdRp) which transcribes their genome and produces mRNAs that can be translated by the host cellular protein synthesis machinery (Payne, 2017).

**Figure 1:**
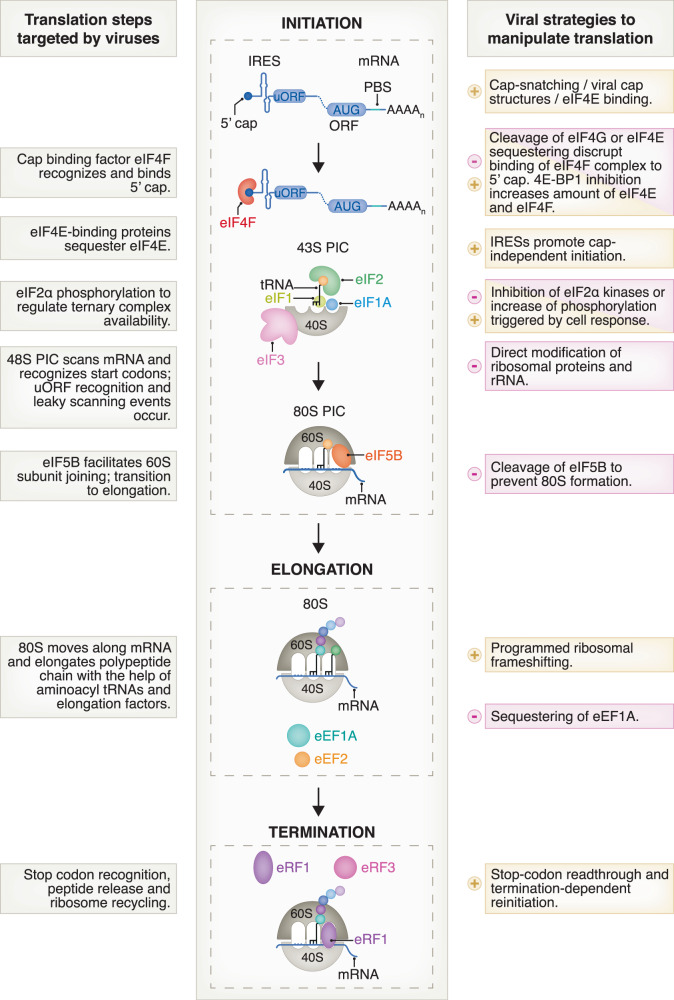
Different viral strategies target translation at different steps. Viruses must use cellular machinery to produce their proteins and many strategies to control translation have evolved. Various viral mechanisms inhibit cellular translation steps (indicated with a minus and pink box). On the other hand, viruses can overcome cellular anti-viral immune responses by promoting the translation of their own viral mRNAs through unique strategies (indicated with plus and beige boxes).

Figure 1 summarizes examples of viral mechanisms employed for inhibiting cellular and promoting viral protein synthesis and exemplifies how all stages of translation can be targeted. The list of viral strategies is not exhaustive but illustrates the diversity of evolved viral mechanisms. This review does not cover in detail specific examples of RNA- or protein-based strategies since several excellent reviews that cover these topics have been written (Stern-Ginossar et al, 2019; Jaafar and Kieft, 2019; Jan et al, 2016; Walsh et al, 2013; Walsh and Mohr, 2011).

During translation initiation, certain viruses modify their RNAs to mimic the 5΄ cap structure of cellular mRNAs using their own capping machinery or by cap-snatching (Decroly et al, 2012; Ramanathan et al, 2016). Thereby, viruses can protect their mRNA from cellular exonucleases, allow their mRNAs to be recognized by eukaryotic translation initiation factor 4E (eIF4E), and evade antiviral innate immune response mechanisms that can recognize RNAs lacking the 5΄ cap (Rehwinkel et al, 2010). Many viruses have evolved diverse and unconventional capping mechanisms. While 5΄ cap formation of the host mRNAs occurs co-transcriptionally inside the nucleus, RNA viruses can perform capping in the cytosol. For example, the capping mechanism of the *Betacoronavirus* SARS-CoV-2 genome has recently been identified. The kinase-like nidovirus RdRp-associated nucleotidyltransferase (NiRAN) domain of the non-structural protein 12 (Nsp12) transfers, via an Nsp9 N-terminus-bound intermediate, a monophosphorylated RNA to GDP, forming the GpppA-RNA (Park et al, 2022). Since cellular defense machinery can sense the lack of terminal ribose 2΄ O-methylation modification and the lack of N7 methylation on the guanosine nucleotide (nt) of the mRNA cap and trigger the interferon (IFN) pathway, viruses evade this by encoding dedicated methyltransferases (Kumar et al, 2014; Dong et al, 2012; Paramasivam, 2020). In coronaviruses these enzymes are the N7 methyltransferase Nsp14 and 2’-O methyltransferase Nsp16, which add methyl groups to the GpppA-modified mRNAs to produce the fully functional cap-0 and cap-1 structures (Chen et al, 2009; Bouvet et al, 2010).

Viral proteins are also able to control translation in host cells by reducing the cellular levels of canonical initiation factors, for example, by proteolytically cleaving eIF4G (Poliovirus protease 2A) (Gradi et al, 1998) or sequestering eIF4E (encephalomyocarditis virus, EMCV) (Gingras et al, 1996), resulting in a decreased amount of functional cap-binding eIF4F complex (Sweeney et al, 2014). Through this strategy, viruses such as EMCV and poliovirus ensure that canonical translation initiation is inhibited. In contrast, viral translation is mostly unaltered due to a distinct mode of translation initiation comprising internal ribosomal entry sites (see below). Conversely, some viruses, including Herpes simplex virus (HSV), stimulate eIF4F formation by inactivating cellular translational repressors, such as 4E-binding protein 1 (4E-BP1), which increases the abundance of eIF4E to cope with the increased need for production of viral proteins (Jan et al, 2016; Walsh and Mohr, 2011; Mohr and Sonenberg, 2012).

Furthermore, viruses can promote host mRNA degradation to control gene expression, reduce protein synthesis and evade host immunity (Abernathy and Glaunsinger, 2015; Burgess et al, 2022; Gaucherand and Gaglia, 2022). Viruses induce mRNA decay either directly by encoding their own nucleases or decapping enzymes, or via cellular RNA decay pathways. In addition to mimicking canonical translation initiation signals, some viruses, including poliovirus and EMCV, have evolved structured mRNA elements, internal ribosome entry sites (IRESs), that recruit ribosomal subunits in a 5΄ cap-independent manner (Jaafar and Kieft, 2019; Kieft, 2008; Mailliot and Martin, 2018; Roberts and Wieden, 2018). Since IRES-dependent translation initiation can be independent of eIF2, viral translation is promoted even when cellular translation initiation mechanisms are inhibited, e.g., by eIF2α phosphorylation in response to cellular stress (Burgess et al, 2022; Costa-Mattioli and Walter, 2020).

Although initiation is the most regulated stage of translation, viruses can also regulate translation during the elongation and termination phase, mainly through structured RNA elements within the ORF. Eukaryotic viruses are under evolutionary pressure to minimize their genome size while staying compatible with the monocistronic nature of the eukaryotic translation machinery, during which initiation proceeds via the recognition of a canonical mRNA 5΄ cap (Atkins et al, 2016; Rozman et al, 2022). Consequently, RNA viruses employ leaky scanning, programmed ribosomal frameshifting (PRF), and translation reinitiation to maximize the range of different translational products that can be generated from a single mRNA (Atkins et al, 2016). Through ribosome profiling experiments, several viral transcriptomes have been annotated and shown to possess overlapping ORFs (Stern-Ginossar et al, 2019) including herpesviruses (HCMV, KSHV) (Stern-Ginossar et al, 2012; Arias et al, 2014), *Vaccinia virus* (Yang et al, 2015), EMCV (Napthine et al, 2017; Hill et al, 2021) (Napthine et al 2017, Hill et al 2021) and several coronaviruses (Irigoyen et al, 2016). Coronaviruses, for example, utilize −1 PRF, during which specific RNA elements cause the elongating ribosome to shift into a different reading frame (Caliskan et al, 2015; Dinman, 2012) (see chapter 6.3). Other viruses that employ −1 PRF include *Human immunodeficiency virus 1* (HIV-1) (Brierley and Dos Ramos, 2006) and West Nile Virus (Melian et al, 2014). Stop codon readthrough is another strategy that increases coding capacity, which generates a longer protein isoform and is signaled by mRNA sequences in the vicinity of the stop codon (Firth and Brierley, 2012). Furthermore, viral proteins are frequently synthesized as polyproteins and subsequently cleaved into mature proteins by dedicated viral proteases (Modrow et al, 2013).

## Evolution of *Betacoronavirus*, genome structure, and function

*Coronaviridae* is a family of enveloped, positive sense single-stranded RNA (+ssRNA) viruses that can infect a broad range of animals, including birds, amphibians, and mammals (Cavanagh, 1997). They are further divided into three subfamilies, the *Letovirinae*, *Pitovirinae* and *Orthocoronavirinae*, the latter known as the coronaviruses. Coronaviruses cause a spectrum of mild to serious and sometimes highly lethal respiratory tract diseases. They are separated into four genera, alpha to delta, of which *Betacoronavirus* is known to infect mammals. Of the five β-CoV subgenera (*Embecovirus, Nobecovirus, Hibecovirus, Sarbecovirus* and *Merbecovirus*), rodents are the natural reservoir for *Embecovirus* and bats for the other four subgenera.

Whereas certain human coronaviruses such as HCoV-HKU1 (Woo et al, 2005) and HCoV-OC43 (Vijgen et al, 2005), belonging to *Embecovirus*, have been circulating among humans since the 1960s and causing common cold symptoms, members of *Sarbeco*- and *Merbecovirus* were in recent years identified as responsible for infections with more severe symptoms (Corman et al, 2018). Among these, severe acute respiratory syndrome coronavirus 2 (SARS-CoV-2) led to the outbreak of the COVID-19 pandemic with a major impact on the world’s health system, as well as on the economic and social order (Gorbalenya et al, 2020). Furthermore, SARS-CoV and the Middle East respiratory syndrome-related coronavirus (MERS-CoV) were responsible for two previous epidemics in 2003 and 2012 respectively (Hilgenfeld and Peiris, 2013). Through spillovers from bats or rodents, first to intermediate hosts such as civets or dromedary camels and then to humans, highly transmissible coronaviruses can rapidly spread in the human population.

Among single-stranded RNA viruses, coronavirus genomes have some of the largest genomes, in some cases exceeding 30 kb (Campillo-Balderas et al, 2015). The genome has a 5΄ cap, harbors a highly structured 5΄ untranslated region (UTR), followed by at least 13 protein-encoding open reading frames, and ending with the 3΄ UTR and a poly(A) tail (Wu et al, 2020; Zhu et al, 2020). The non-structural proteins (Nsps), important for viral RNA replication transcription and translation, are encoded as a polyprotein within the ORF1ab (translated as polyproteins pp1a and pp1ab as a consequence of a translational frameshifting event discussed later) in the 5΄ proximal region of the genomic RNA. The two virus-encoded proteases, papain-like (PL^Pro^, Nsp3) and chymotrypsin-like or main (3CL or M^Pro^, Nsp5) protease, cleave the polyproteins pp1a and pp1ab, liberating the Nsps (Snijder et al, 2003). The first protein to be synthesized and released is Nsp1 and its role is to shut down host mRNA translation. In contrast, all the other non-structural proteins, besides Nsp2, are responsible for remodeling the cellular membranes (Nsp3, Nsp4 and Nsp6) or constitute components of the RNA replication and transcription machinery (Nsp5, Nsp7-16) (Snijder et al, 2016).

Upstream open reading frames (uORFs) are usually short ORFs present in the 5΄ UTR that are often found in the leader sequences of eukaryotic transcripts. While their function is largely unclear, they often repress translation of the main ORF (Calvo et al, 2009). In other cases, they enhance the main ORF translation through different mechanisms or stimulate translation-dependent mRNA degradation (Dever et al, 2023; Wek, 2018; Karousis and Mühlemann, 2022). uORFs are also found in the genomic 5΄ UTR of most coronaviruses (Wu et al, 2014). Reverse genetics studies using the mouse hepatitis coronavirus showed that uORF disruption can lead to minor changes in virus behavior, but over time the virus often reverts or adapts to restore the presence of a uORF. These adaptations suggest that the uORF, while not essential, is translated and supports optimal virus replication in cell culture (Wu et al, 2014; Irigoyen et al, 2016). In case of SARS-CoV-2, although several initiation sites in the SARS-CoV-2 5′ UTR have been detected (Finkel et al, 2021b), it is unclear to what extend they affect translation of the viral genome.

## Coronaviruses modulate host cell translation

The term “host shutoff” has been coined to describe the selective inhibition of cellular protein synthesis upon viral infection. This inhibition increases viral access to translation resources and reduces the capacity of the host cell to mediate antiviral responses such as the production of interferons, proteins that signal viral presence and promote the antiviral defenses of neighboring cells (Rozman et al, 2022; de Breyne et al, 2020). Viral mRNAs, in turn, have unique features that allow them to bypass translation inhibition.

**Figure 2:**
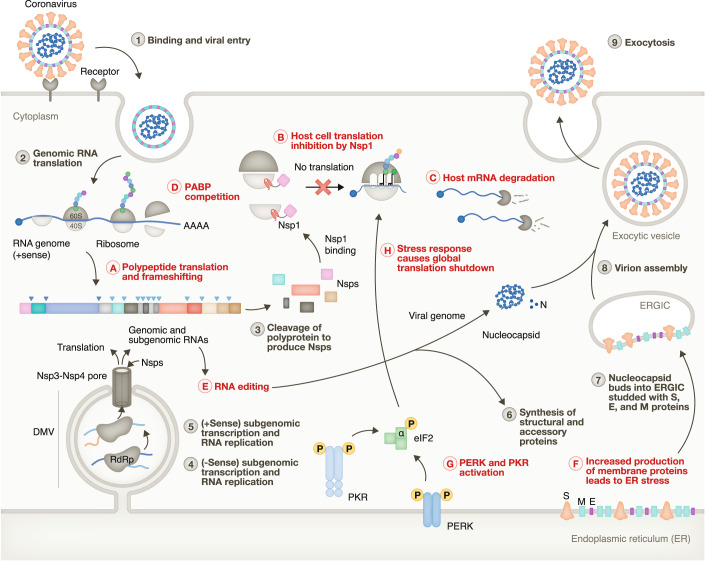
Life cycle of coronaviruses and implications for translation regulation. Numbers (1–9) indicate life cycle steps of the virus, while interactions with host translation are represented by letters (A–H). Upon binding of a virion to host receptors, membrane fusion releases the positive sense RNA genome into the cytoplasm (1). The genome is translated by the host cell machinery to produce two different polyproteins by programmed ribosomal frameshifting (2 and A). Cleavage of polypeptides (3) produces non-structural proteins (Nsps), including RdRp that synthesize negative and positive sense (−Sense and +Sense) RNAs (4, 5) inside of double-membrane vesicles (DMV) that act as platforms for viral replication. Nsp1 binds to ribosomes (B), inhibits host mRNA translation and stimulates host mRNA degradation (C). Viral RNAs are polyadenylated, and when their concentration is increased, they compete to bind Poly(A)-binding proteins (PABP) (D). RNA editing (E) may introduce variable sequences in the viral progeny and alter the translation and stability properties of the viral RNAs. The subgenomic RNAs are translated to synthesize structural and accessory proteins (6 and F) that can stimulate stress responses (G) in the ER. PKR and PKR-like endoplasmic reticulum kinase (PERK) activation triggers eIF2α phosphorylation that, in turn, inhibits cap-dependent translation (H). The nucleocapsid buds into the ER–Golgi intermediate compartment (ERGIC), which is covered by the structural proteins S (spike), E (envelope), and M (membrane) (7). Exocytosis (8 and 9) exports the virion from the cell.

Coronavirus infection begins when the virions bind cellular receptors, resulting in fusion or endocytosis of the virus and the release of the nucleocapsid genome into the cytoplasm of the host cell (Fig. 2). At this point, the positive sense viral genome functions as an mRNA and is translated to produce non-structural proteins, including replicase, the viral RNA-dependent RNA polymerase. The first ORF (ORF1) is translated into a polyprotein that is proteolytically cleaved to yield several non-structural proteins. Programmed −1 ribosomal frameshifting (on a *cis* RNA element) allows the generation of two different forms of the polyprotein that leads to the generation of two alternative ORF1a and ORF1ab polyproteins. A minus-strand replicative intermediate RNA is used as a template to synthesize plus-strand subgenomic RNAs (sgRNAs), which mainly encode structural proteins required to pack the genome into new viral particles (de Breyne et al, 2020; V’kovski et al, 2021).

### Coronaviral infections trigger stress response mechanisms

Coronaviral infections activate stress responses in different ways. The production of dsRNA during viral replication and the increased abundance of highly structured viral RNAs can trigger PKR activation that, in turn, leads to eIF2α phosphorylation and translation shutdown (Fig. 2). Additionally, the production and maturation of all coronaviral structural proteins, apart from the N protein, occur in the ER. Such increased membrane protein load exceeding the functional capacity of the ER activates the unfolded protein response (UPR), which includes, among others, PERK activation (de Breyne et al, 2020; Stukalov et al, 2021; Shajahan et al, 2020; Perrier et al, 2019).

In the context of viral infections, the outcome of cellular stress varies among different coronaviruses and tissues. For instance, due to the presence of the dsRNA, SARS-CoV-2 triggers PKR and the IFN-regulated RNase L to different extents among different cell types (Li et al, 2021). However, other coronaviruses can also shut down both pathways, as shown for MERS-CoV and Mouse Hepatitis Virus (MHV) (Ye et al, 2007; Comar et al, 2019). ER stress resulting from SARS-CoV or MERS infections suggested that PERK activation, rather than PKR, leads to eIF2α phosphorylation (Krähling et al, 2009; Chan et al, 2006; Versteeg et al, 2007). Furthermore, it was shown that the production of SARS-CoV-2 Spike glycoprotein (S) on its own can trigger UPR, which was also observed after SARS-CoV-2 infection of both Vero and Calu3 cells (Echavarría-Consuegra et al, 2021). Overall, UPR may be an attractive therapeutic target (Upadhyay and Gupta, 2022) because its pharmacological inhibition reduces the replication of MHV and SARS-CoV-2 (Echavarría-Consuegra et al, 2021).

Translational shutdown due to eIF2α phosphorylation stimulates the formation of stress granules and P-bodies that facilitate mRNA storage for later translation or degradation. The Nucleoprotein from SARS-CoV-2 can be incorporated into stress granules either when overexpressed (Savastano et al, 2020) or during viral infection (Li et al, 2022). Its presence in stress granules resistant to disassembly after stress alleviation explains the accumulation of SARS-CoV-2 proteins in the brain tissues of mice and patients who died from COVID-19 (Song et al, 2021). How the Nucleoprotein-mediated stabilization of stress granules affects bulk translation in neurons remains unclear.

Coronaviruses have also been implicated in activating additional signaling pathways, such as the p38 mitogen-activated protein kinases (MAPKs) and extracellular signal-regulated kinase (ERK) with effects on the host cell translation activity (de Breyne et al, 2020). Upon SARS-CoV infection, activated p38 MAPKs phosphorylate eIF4E to attenuate protein synthesis, but the viral replication and protein synthesis were not affected by p38 MAPK-targeting molecules (Padhan et al, 2008; Kopecky-Bromberg et al, 2006). Likewise, SARS-CoV-2 phosphoproteomic data suggests the activation of kinases from p38 MAPK and ERK pathways (Bouhaddou et al, 2020). ERK activation causes eIF4E-binding proteins phosphorylation, which prevents eIF4E from binding to the cap and forming productive eIF4F complexes. As a result, translation is downregulated (Proud, 2019). SARS-CoV, MERS-CoV, and HCoV-229E infections, and overexpression of the SARS-CoV S protein led to ERK activation (Liu et al, 2007; Mizutani et al, 2004; Ghasemnejad-Berenji and Pashapour, 2021; Bouhaddou et al, 2020).

### How Nsp1 hijacks cellular translation

The first Nsp in genomes of many coronaviruses is the pathogenicity factor Nsp1 (Kamitani et al, 2006; Wathelet et al, 2007; Züst et al, 2007). Nsp1 proteins are present in *Alpha*- and *Betacoronavirus*, but they can differ in size, from 110 to 245 amino acids (Sosnowski et al, 2022; Nakagawa and Makino, 2021), and in sequence. Despite these differences, Nsp1 induces translational shutdown and viral evasion in all studied viruses including SARS-CoV, MERS-CoV, transmissible gastroenteritis virus (TGEV) and MHV (Kamitani et al, 2006; Terada et al, 2017; Wang et al, 2010; Shen et al, 2019; Brockway and Denison, 2005). However, the exact translation suppression mechanism may vary for each virus (Tidu et al, 2021) since, interestingly, *Gammacoronavirus* and *Deltacoronavirus* do not encode Nsp1. In these viral subgroups, exemplified by the avian infectious bronchitis virus (IBV), a *Gammacoronavirus*, it has been proposed that the accessory protein 5b takes over the role of Nsp1 to induce host shutoff (Kint et al, 2016).

The host shutoff mechanism has been extensively studied in β-CoVs, especially in SARS-CoV (Kamitani et al, 2006; Wathelet et al, 2007; Narayanan et al, 2008), MERS-CoV (Lokugamage et al, 2015) and most recently in SARS-CoV-2. SARS-CoV and SARS-CoV-2 Nsp1 are small 180-amino acid proteins with two domains—an N-terminal domain (NTD) connected via a linker to the C-terminal domain (CTD). Mutational studies in SARS-CoV Nsp1 have identified residues K164 and H165 in the CTD (“KH motif”) as being critical for translation inhibition. The first structural characterization of SARS-CoV Nsp1 using nuclear magnetic resonance (NMR) spectroscopy, however, revealed the structure of the NTD only (Almeida et al, 2007). Later studies in SARS-CoV-2 confirmed the fold of the NTD and highlighted the flexible nature of the C-terminal region (Clark et al, 2020; Semper et al, 2021; Kumar et al, 2021a).

**Figure 3:**
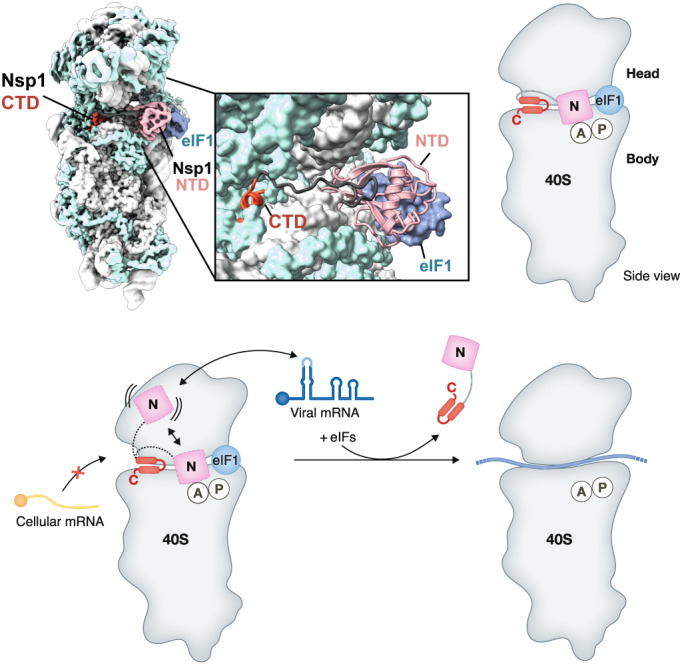
Nsp1 inhibits translation by binding to the mRNA channel (through its CTD) and the A-site of the 40S (through its NTD). The CTD binds with high affinity to the mRNA entry channel, where it anchors Nsp1 to the ribosome and would sterically clash with mRNA accommodation. The NTD dynamically interacts with the decoding center (ribosomal A-site), further contributes to translation inhibition, and plays a role in the selective translation of viral mRNAs.

Since the outbreak of SARS-CoV-2, investigation of the mechanism of translation inhibition by coronaviruses has received increased attention. As a result, cryo-electron microscopy (cryo-EM) structures revealed that Nsp1 CTD folds into two helices as it binds to the mRNA entry channel of the small ribosomal subunit, where it would sterically clash with the mRNA (Fig. 3) (Schubert et al, 2020; Thoms et al, 2020; Yuan et al, 2020; Lapointe et al, 2021). The conserved KH motif interacts with helix h18 of the 18S ribosomal RNA (rRNA) to position the CTD of Nsp1 inside the mRNA channel, which would prevent mRNA accommodation and inhibit translation. This mode of action seems to be conserved among β-CoVs since Nsp1 from MERS-CoV and from a β-CoV that infects bats, *Bat Hp betacoronavirus Zhejiang2013* (Bat-Hp-CoV), also binds to the 40S and suppresses translation (Fig. 3) (Schubert et al, 2023). Although Nsp1 shuts down protein synthesis by binding to the 40S ribosomal subunit, biochemical data suggests that viral subgenomic and genomic mRNAs are efficiently translated despite Nsp1 inhibition (Mendez et al, 2021; Banerjee et al, 2020; Bujanic et al, 2022). The leader sequence in the 5΄ UTR, which is present in the genomic RNA (gRNA) and all sgRNA, is crucial for viral evasion of the translational shutdown. Three nts within the first stem-loop SL1 (C15, C19, and C20) were biochemically shown to be critical for this evasion mechanism (Bujanic et al, 2022). Additionally, mutational studies have shown that mutations R124A/K125A and R99A in the Nsp1 NTD prevented the selective translation of viral mRNAs, indicating an active role of Nsp1 in selectively allowing viral mRNAs to form productive translational complexes (Mendez et al, 2021). Furthermore, clinically relevant Nsp1 variants have emerged: ΔKSF Nsp1 with a deletion in the linker (residues 141–143 in SARS-CoV-2) (Benedetti et al, 2020), V121D Nsp1 present in SARS-CoV-2 strain NIB-1 (Hossain et al, 2021) as well as Δ500-532 Nsp1 with a deletion in the NTD (residues A79-V89 in SARS-CoV-2) (Lin et al, 2021). These variants all have in common functional impairment of the NTD, for example, through protein misfolding. Moreover, these variants correlate with reduced pathogenicity traits compared to SARS-CoV-2 wild-type and consequently lower levels of IFN-β production (Lin et al, 2021).

A recent structural and biochemical study explained how viral mRNAs are preferentially translated in the presence of Nsp1 (Schubert et al, 2023) (Fig. 3). The NTD of Nsp1 was, for the first time, observed interacting with the ribosome bound to the decoding center on the 40S subunit, where it would additionally interfere with mRNA accommodation and contribute to translation inhibition. Although the structural data was only obtained for the Bat-Hp-CoV, structure-based mutagenesis suggests that this transient interaction also exists in other species of *Betacoronavirus*. Interestingly, the residues of Nsp1 that mediate 40 S binding are the same ones that are critical for allowing viral RNA translation (Bujanic et al, 2022; Mendez et al, 2021; Schubert et al, 2023). Consequently, viral RNAs, with their unique 5΄ UTRs, may be preferentially translated by interfering with the accommodation of the N-terminal domain into the decoding center, thereby reducing the inhibitory potential of Nsp1. A recent study proposed that in solution, the CTD tail interacts with a positively charged surface patch within the NTD (Wang et al, 2023). Such an interaction would protect the CTD from degradation and ensure the viral mRNA is only recruited once Nsp1 is attached to the ribosome, explaining why free Nsp1 does not bind viral mRNA.

Further studies are required to understand whether Nsp1 remains attached to the ribosome during viral translation or dissociates before mRNA scanning and translation elongation. Two models exist, one proposing that Nsp1 remains attached to the ribosome during viral translation (Tidu et al, 2021), whereas the other model states that Nsp1 needs to dissociate for viral mRNA to accommodate in the mRNA channel (Banerjee et al, 2020). Another study has also shown that 5΄-terminal oligo-pyrimidine (TOP) host mRNAs are preferentially translated in the context of Nsp1 expression (Rao et al, 2020). So far, the molecular mechanism and the corresponding *cis*-elements within the 5΄ UTRs of TOP mRNAs remain uncharacterized (Rao et al, 2020; Eriani and Martin, 2022). However, it may be possible that unique sequence features of these RNAs interfere with the accommodation of the NTD onto the decoding center according to the same mechanism proposed for the viral RNAs. From a viral perspective, such an evasion would make sense since TOP mRNAs encode ribosomal proteins and translation factors and are therefore required to maintain functional protein synthesis machinery to produce viral proteins.

Inhibition of cellular mRNA translation by SARS-CoV-2 Nsp1 prevents the synthesis of IFNs and other pro-inflammatory cytokines as well as IFN-stimulated anti-viral mRNAs (Thoms et al, 2020). In agreement with this observation, viral replicons with Nsp1 mutants were more sensitive to interferon-α (IFNα) and interferon-β (IFNβ) compared with WT replicons (Ricardo-Lax et al, 2021). Lastly, infection experiments showed that the functional importance of SARS-CoV-2 infection relies on blocking the IFN response (Fisher et al, 2022). The inhibition of IFN responses by Nsp1 contributes to the pathogenesis of SARS-CoV-2 (Kim and Shin, 2021), allowing the virus to evade the host’s innate immune defenses, especially in the early stages of infection.

In addition to inhibiting protein synthesis by binding to the ribosome, additional roles of Nsp1 in host mRNA decay and export have been proposed. Early reports on SARS-CoV have shown that Nsp1 promotes host mRNA cleavage and degradation while sparing viral mRNAs (Narayanan et al, 2008; Kamitani et al, 2009; Huang et al, 2011; Narayanan et al, 2015). According to this model, Nsp1 induces endonucleolytic RNA cleavage in the 5΄ UTR of cellular mRNAs, which are subsequently degraded by the Xrn1-mediated 5΄–3΄ exonucleolytic mRNA decay pathway (Gaglia et al, 2012; Narayanan et al, 2015). More recent studies in SARS-CoV-2 have shown an accelerated global degradation of host mRNAs during the early stages of viral infection, arguing for a viral takeover of the mRNA pool (Burke et al, 2021; Finkel et al, 2021a). Furthermore, mRNA degradation seems to occur co-translationally (Mendez et al, 2021) and might require additional translation or *trans*-acting factors. Since Nsp1 harbors no intrinsic nuclease activity, and the acting host endonuclease remains to be characterized, further studies are required to uncover the molecular mechanism of Nsp1-mediated mRNA degradation. It has also been proposed that Nsp1 also affects the nuclear export of cellular mRNAs by interacting with the mRNA export receptor NXF1-NXT1, thereby preventing NXF1 from engaging with the nuclear pore complex to mediate mRNA translocation (Zhang et al, 2021).

Interestingly, Nsp1 has been described to target the DNA polymerase α (Pol α)-primase complex, potentially interfering with IFN-related signaling (Kilkenny et al, 2022). Besides Nsp1, Nsp2 has also been described to mediate host shutoff in a translation-related manner. Through associating with the translation inhibitor complex 4EHP-GIGYF2, the type I IFN-β translation is downregulated, impairing the immune response pathway and leading to evasion of the immune response (Gordon et al, 2020; Gupta et al, 2021; Xu et al, 2022).

In summary, the role of Nsp1 in host translation shutdown seems to be the most firmly established function among all Nsps. During early infection, Nsp1 is expressed to inhibit protein synthesis by blocking ribosomes. While host mRNA translation is inhibited, viral mRNAs evade this inhibition. Future structural, biophysical, and biochemical studies are required to complete the mechanistic framework of Nsp1-mediated viral evasion. Such a translation inhibition mechanism mediated by a viral protein is unique among all viruses. Interestingly though, the endogenous hibernation factor SERPINE mRNA-binding protein 1 (SERBP1) also binds to the mRNA channel, prevents mRNA binding, and sequesters 80S ribosomes (Brown et al, 2018; Anger et al, 2013).

## Unique features of CoV-encoded RNAs

### Structural features of viral RNAs

Single-stranded viral RNAs, through their ability to adopt complex folds, play important roles in regulating the viral life cycle with crucial implications in translation, replication, and transcription (V’kovski et al, 2021). Upon SARS-CoV and MERS-CoV infections, viral mRNAs robustly evade translation inhibition, whereas host cell mRNAs are largely affected. The capacity of viral mRNAs to replicate and withstand translation inhibition heavily relies on regulatory elements harbored in viral mRNAs (Banerjee et al, 2020; Nakagawa et al, 2016). Consequently, viral protein production is sustained at high levels.

**Figure 4:**
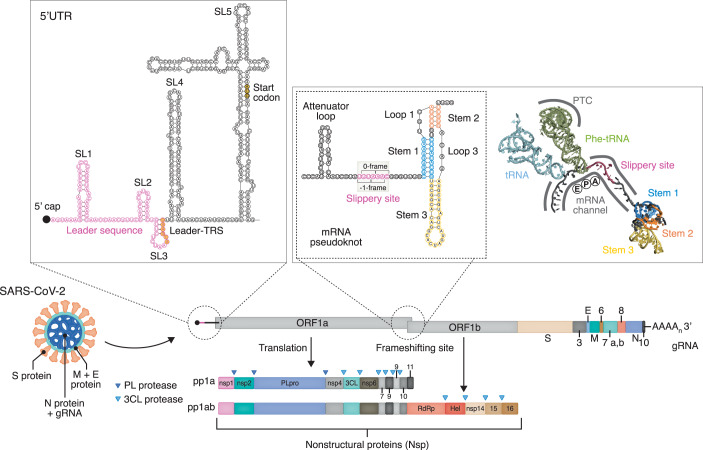
Features of coronaviruses to hijack and regulate translation in host cells. In −1 programmed ribosomal frameshifting (upper right box), the ribosome encounters a frameshifting site, upon which the ribosome shifts its frame towards −1 within the slippery site. This is caused by the pseudoknot that resides at the mRNA entry channel (PDB: 7O7Z). Upon infection, translation is inhibited by Nsp1 through binding to the 40S subunit. Viral mRNAs harboring the first stem-loop (SL1) in their 5΄ UTR (upper left box) evade Nsp1-mediated inhibition. The mRNA secondary structure of the 5΄ UTR is based on Wacker et al (2020) and Miao et al (2021) and the mRNA pseudoknot on Bhatt et al (2021).

Coronaviruses have particularly long RNA genomes, ranging between 26 and 32 kb, that are 5΄-capped and 3΄-polyadenylated. The most characteristic functional regulatory elements are found in the 5΄ and 3΄ UTRs. Several stem-loops in the 5΄ UTR (nts 1-265 in SARS-CoV-2) are major RNA structural elements and important for viral replication (SL1 and SL2), subgenomic RNA production (SL3 and SL4) and escape from Nsp1-mediated translational repression (SL1). The start codon is harbored in the well-folded SL5, which branches in three separate loops (SL5A, SL5B and SL5C) (Fig. 4) (Huston et al, 2021; Lan et al, 2022). Structural elements in the 3΄ UTR are more variable among different coronaviruses (Madhugiri et al, 2016).

All coronaviral transcripts harbor common leader sequences ranging between 60 and 95 nt in length, and the formation of characteristic secondary and tertiary structures is essential for functional RNA-RNA and RNA-protein interactions during different stages of the viral lifecycle (V’kovski et al, 2021). Transcription-regulating sequences (TRSs) are required to synthesize sgRNAs by the replication-transcription complex (RTC) and control the joining of the leader to the body of the subgenomic mRNA. TRSs are located downstream of the 5΄ leader (leader-TRS or TRS-L) and upstream of the ORFs in the 3΄-proximal region of the gRNA (body-TRS or TRS-B) (Woo et al, 2023). Dimethyl sulfate (DMS) mutational profiling with sequencing (DMS-MaPseq) of the SARS-CoV-2 RNA genome from infected cells showed that the structures adopted by TRS-L vary in different sgRNAs depending on the neighboring sequence context. Also, the structural features correlate with the abundance of the corresponding RNAs (Sun et al, 2021b; Tavares et al, 2021). Additional RNA sequences containing stem-loops (packaging signals) are required for genome packaging and are thoroughly discussed elsewhere (Madhugiri et al, 2016).

In SARS-CoV-2, SL1 in the 5΄ UTR as well as the frameshifting element between ORF1a and ORF1b play important roles in modulating gRNA translation (Fig. 4) (V’kovski et al, 2021; Madhugiri et al, 2018).

SL1 is necessary and sufficient for bypassing Nsp1-mediated translation inhibition during SARS-CoV-2 infections (Vora et al, 2022; Banerjee et al, 2020). SL1 is particularly conserved as there are no known single nt variants with >1% frequency, implying a crucial functional role. The 5΄ leader must be precisely positioned relative to the 5΄ cap to allow translation initiation in the presence of Nsp1 (Banerjee et al, 2020). Furthermore, the functional interaction between SL1 and the NTD of Nsp1 may play an important role in the selective translation of viral RNAs (Schubert et al, 2023). Consistent with this role, antisense oligos targeting SL1 could suppress viral translation by reducing the translation efficiency of the viral 5΄ UTR and by inhibiting evasion of viral 5΄ leader-containing mRNAs from Nsp1-mediated suppression (Bujanic et al, 2022). The therapeutic potential of such oligos is supported by their inhibition of SARS-CoV-2 replication in cell culture and decreased lethality of SARS-CoV-2 infections in mice (Vora et al, 2022).

The 5΄ UTR present in the SARS-CoV-2 gRNA, but not in the sgRNAs, is highly structured, especially because of the particularly stable SL5 (Miao et al, 2021). Mechanistic and structural insights are required to address the function of SL5 and the unfolding mechanism that would allow a scanning-dependent translation initiation for gRNAs.

Apart from the highly conserved elements in the 5΄ and 3΄ UTRs, recent advances in high-throughput chemical RNA probing methods have revealed structural features of the total genome. Ex vivo extracted and refolded SARS-CoV-2 RNA provided single base resolution secondary structure maps of the full genome and revealed that, in many cases, there is heterogeneity in the formed secondary structures (Manfredonia et al, 2020). DMS-MaPseq on SARS-CoV-2-infected cells confirmed the structural heterogeneity of the RNA, highlighting regions that fold into single structures with functional implications, especially concerning the frameshifting site (Lan et al, 2022). The complete secondary structure of the SARS-CoV-2 genome from living cells obtained by SHAPE-MaP (selective 2′-hydroxyl acylation analyzed by primer extension-based mutational profiling) revealed elaborate networks of well-folded, secondary structure elements present across coding sequences, a characteristic feature that is distinct from other positive-sense RNA viruses with smaller genomes (Huston et al, 2021). The exceptionally long SARS-CoV-2 genome contains fewer long-range base pairing interactions compared to other positive-sense RNA viruses, a feature that facilitates the evasion from innate immune responses, preserves translation fidelity, maintains genomic stability, and possibly evades phase separation at high viral RNA concentrations (Huston et al, 2021; Tavares et al, 2021). Well-folded regions of viral RNA located at protein domain boundaries may reduce the speed of translocating ribosomes to promote domain-wise protein folding, co-translational assembly, and processing.

### Host proteins can bind and affect viral RNAs

Viral RNAs bear several features that mimic host mRNAs (such as 5΄ caps and poly(A) tails) and have evolved secondary structures that recruit cellular factors and complexes that enhance robustness and expression efficiency, often in a translation-dependent manner. Viral RNAs also present themselves as substrates for cellular RNA editing enzymes to diversify their coding potential and can recruit protein complexes that affect the fate of viral RNAs in cells.

#### RNA editing

Genetic variations that occur in the viral RNA genome due to errors during RNA synthesis, recombination and shuffling of RNA segments, and RNA editing (V’kovski et al, 2021) may lead to the emergence of new strains. RNA editing of viral genomes increases genetic variability as a starting point for further natural selection (Mourier et al, 2021). ADARs (adenosine deaminases that act on RNA) target dsRNA and deaminate adenines into inosines (A-to-I), and APOBECs (apolipoprotein B mRNA editing catalytic polypeptide-like proteins) deaminate cytosines into uracils (C-to-U) on ssDNA and ssRNA (Rengaraj et al, 2021). Evidence shows that these two classes of mammalian enzymes edit SARS-CoV-2 RNAs during infection.

Early results from mutational analysis of genomes from different strains of *Betacoronavirus* from human hosts suggested that the activity of both APOBECs and ADARs may lead to the restriction of viral propagation (Di Giorgio et al, 2020). However, an increased rate of A-to-G substitutions has been observed in minor SARS-CoV-2 RNA populations in patients with decreased viral loads. This implies that over time, ADARs may indeed give rise to new variants of SARS-CoV-2 with different infectivity and transmissibility (Ringlander et al, 2022). SARS-CoV-2 patient-derived sequences also revealed an increased C-to-U mutation incidence. This observation was linked to APOBEC3A, APOBEC1, and APOBEC3G, however, viral replication and progeny production were not inhibited by the expression of these APOBECs (Kim et al, 2022). A link between RNA editing and the production of new SARS-CoV-2 variants could help predict viral genome mutations, facilitating the identification of genomic hotspots for therapeutic interventions and RNA design (Kim et al, 2022). Genetic polymorphisms of APOBEC3A and APOBEC3B in different populations (Kidd et al, 2007) could also influence the spread of infections (Di Giorgio et al, 2020). Considering that RNA modifications modulate translation efficiency (Ranjan and Leidel, 2019; Kidd et al, 2007), it will be interesting to explore how viral RNA editing impacts translation dynamics.

#### Poly(A) binding proteins

Mammalian mRNAs possess, on average, a ~200 nt poly(A) tail that regulates mRNA translational status and stability. Cytoplasmic poly(A) tails are bound by PABPs. Cytoplasmic PABP (PABPC1 in humans) requires approximately 12 adenosines for stable binding and covers approximately 30 nts. Longer tails can, in principle, bind more PABPs, but the degree of saturation may depend on the concentration of PABP in the cytoplasm (Passmore and Coller, 2022).

SARS-CoV-2 RNAs carry ~47 nt poly(A) tails, and the length is considerably longer for genomic compared to subgenomic RNAs (Kim et al, 2020). In SARS-CoV and SARS-CoV-2, a region of Nsp3 protein that forms pores in the double membrane vesicles in infected cells, named SARS-unique domain (SUV) interacts with the PABP-interacting protein 1 (Paip1). The interaction of Nsp3 with Paip1 enhances viral and reduces host cell translation in SARS-CoV and SARS-CoV-2, but not in MERS-CoV, by an unknown mechanism (Lei et al, 2021). It has been reported for bovine (BCoV) and mouse hepatitis CoVs (MHC) that binding of the PABP to the poly(A) tail of the viral RNA is required for viral replication (Spagnolo and Hogue, 2000). BCoV Nucleoprotein promotes genome circularization that allows negative-strand RNA synthesis, mimicking the interaction between eIF4G and PABP that occurs during canonical translation (Lo et al, 2019).

#### Cap-binding proteins

The presence of a 5΄ cap on viral RNAs implies that translation initiation is cap-dependent and proceeds via the participation of canonical initiation factors. In line with this notion, downregulation of the eIF4A1 RNA helicase from the cap-binding eIF4F complex reduced the expression of SL1-containing reporter mRNAs in vivo (Slobodin et al, 2022). Several studies have shown that molecules and compounds that interfere with the interactions between eIF4F components inhibit viral protein synthesis or viral replication in HCoV-229E (Cencic et al, 2011) and MERS-CoV (Müller et al, 2018). As expected for a cap-dependent initiation mechanism, translation of the genomic RNA of SARS-CoV-2 containing the entire 5΄ UTR requires all components of the eIF4F complex (Condé et al, 2022). Emetine, a eukaryotic translation inhibitor which is an FDA-approved drug for anti-protozoal treatment, disrupts the binding of SARS-CoV-2 mRNA to the cap-binding protein eIF4E, suppressing viral replication (Kumar et al, 2021b). However, seemingly contradictory results indicated that a cap-independent translation of the full-length 5΄ UTR is possible since translation was observed in the presence of Torin1 inhibitor that limits eIF4E availability via the phosphorylation of eIF4E-binding protein (Slobodin et al, 2022).

### Programmed −1 ribosomal frameshifting in *Betacoronavirus*

A key event during the translation of the viral ORF1ab is −1 programmed ribosomal frameshifting (-1 PRF), a translational recoding event. The PRF recoding site contains three main mRNA elements: the slippery sequence, a downstream secondary structure element (pseudoknot in coronaviruses), and a spacer connecting the slippery sequence and pseudoknot (Fig. 4). It has been proposed that once the ribosome encounters the pseudoknot mRNA fold during elongation, translation slows down, and the ribosome resides longer than usual on the slippery sequence, which at this point is located in the decoding center (Brierley et al, 1989; Chen et al, 2020; Choi et al, 2020; Riegger and Caliskan, 2022). Within this time window, some of the ribosomes shift their reading frame by one nt, and translation proceeds in the new −1 frame (Brierley et al, 1989; Su et al, 2005). In most cases, the slippery sequence is X_XXY_YYZ (0 frame), in which X can be any nt, Y either adenine or uridine, and Z any nt besides guanine (Riegger and Caliskan, 2022; Brierley et al, 1989; Dinman et al, 1991).

Recently, a combination of structural, biochemical, and molecular dynamics studies have elucidated the molecular mechanism of PRF in SARS-CoV-2 (Bhatt et al, 2021; Roman et al, 2021; Jones and Ferré-D’Amaré, 2022; Napthine et al, 2021; Zimmer et al, 2021). A cryo-EM study revealed that the stimulatory pseudoknot structure resides at the ribosomal mRNA entry channel and specifically interacts with ribosomal proteins uS3 and eS10. This interaction prevents pseudoknot unfolding at the entry into the mRNA channel by the positively charged ribosomal proteins with intrinsic helicase activity (Rabl et al, 2011). Based on this observation, it has been proposed that the lodged pseudoknot resists unfolding during elongation factor-stimulated translocation, thereby promoting tension within the mRNA to stimulate −1 frameshifting within the slippery site (Fig. 4, right box) (Bhatt et al, 2021). Ribosome profiling experiments revealed that ribosome collisions occur with the leading ribosome at the same position where the pseudoknot-engaged ribosome was trapped in the cryo-EM structure, indicating ribosomal pausing just before the frameshifting event (Bhatt et al, 2021). Lastly, it was observed that the nascent viral polyprotein may influence frameshifting efficiency by specifically interacting with the ribosomal exit tunnel (Bhatt et al, 2021).

PRF is conserved among all coronaviruses, and this process is critical for synthesizing Nsp12, the catalytic component of the RdRp encoded by both the ORF1a and the frameshifted ORF1b. Coronaviruses, and other viruses, such as HIV, use programmed −1 frameshifting to expand their genomic coding capacity as well as fine-tune the stoichiometric ratio of expressed viral proteins (Plant et al, 2010). With an estimated frameshifting efficiency of 45 to 70% in the case of SARS-CoV-2, this results in a stoichiometric ratio of 1.5–2-fold excess of ORF1a proteins over ORF1b proteins (Finkel et al, 2021a, 2021b). Furthermore, −1 PRF plays a role in the timing of events during the viral life cycle. During early infection, mainly the non-structural proteins from the ORF1a are produced in the cell to inhibit host innate immune response pathways. At a later stage of the viral replication cycle, with more frameshifting events, the viral replicase is produced to allow increased RNA synthesis (Kelly et al, 2021).

In addition to *cis*-acting elements within the viral RNA, PRF is also modulated by host *trans*-acting factors. Among these, the host cell factors shiftless (SFL), induced by the IFN cell signaling pathway, and a short isoform of the zinc-finger antiviral protein (ZAP), have been identified. Both SFL and ZAP have been described to interact with ribosomes during frameshifting, to alter the efficiency of the frameshifting and disadvantage the virus (Wang et al, 2019, Napthine et al 2021, Schmidt et al, 2020, Yang and Li 2020, Zimmer et al 2021). However, it is unclear how these proteins bind to and act on the ribosome. Furthermore, several compounds have been predicted by computational modeling to bind the pseudoknot, and some of them can perturb the frameshifting efficiency (Kelly et al, 2020; Park et al, 2011). Additionally, it has been shown that merafloxacin, a fluoroquinolone compound, inhibits −1 PRF of SARS-CoV-2 in infected cells (Sun et al, 2021a; Bhatt et al, 2021). Future studies are required to fully understand the molecular mechanism of this process, since even small changes in the efficiency of −1 PRF could have a dramatic effect on viral propagation and the viral life cycle.

## Conclusion and outlook

Additional features of coronaviral RNAs are likely to play a role during their translation in infected cells with a wide range of possible physiological implications. For example, leaky scanning, during which a weak initiation codon may be skipped, has been reported in different coronaviruses (Yang et al, 2009; de Breyne et al, 2020) and may be responsible for the production of yet unidentified viral peptides with physiological significance (Finkel et al, 2021a; Kim et al, 2021; de Breyne et al, 2020). Moreover, viral RNA translation may be regulated by uORFs (Finkel et al, 2021a; Kim et al, 2021; de Breyne et al, 2020), adding an additional layer of translation regulation in coronaviral genomes. Lastly, the interplay between coronaviral infections and host cell stress response mechanism remains to be further elucidated.

The striking similarities in the mode of translation inhibition among members of *Betacoronavirus* that rely on characteristic structural features of Nsp1 (Schubert et al, 2023) provide potential points for the structure-based chemical design of inhibitors. However, so far only a few studies that apply structure-based drug discovery have been conducted. One study identified potential pockets for ligand binding using molecular dynamics and fragment-bound Nsp1 NTD crystal structures (Borsatto et al, 2022). According to this study, binding of fragments to these pockets could interfere with the interaction between SL1 in the 5΄ UTR mRNA and Nsp1 and, thereby, inhibit the viral evasion mechanism. Additionally, it was shown that MHV mutants that lack functional Nsp1 are attenuated in mice and were proposed as the basis for a live attenuated vaccine for SARS-CoV (Lei et al, 2013; Züst et al, 2007; Wathelet et al, 2007). Along the same lines, in the SARS-CoV replicon system, mutation of the Νsp1 R124 residue decreased viral gene expression and replication (Tanaka et al, 2012).

Targeting the key functional interactions between the viral components and the host cellular machinery during viral infections provides many possibilities for expanding our arsenal of strategies against coronaviral infections. Despite insightful biochemical and structural data obtained so far, many important questions remain open regarding the unique aspects of viral RNA translation, and how cellular defense systems target this process and viral RNAs.
